# Genetic insights into the regulatory pathways for continuous flowering in a unique orchid *Arundina graminifolia*

**DOI:** 10.1186/s12870-021-03350-6

**Published:** 2021-12-10

**Authors:** Sagheer Ahmad, Chuqiao Lu, Jie Gao, Rui Ren, Yonglu Wei, Jieqiu Wu, Jianpeng Jin, Chuanyuan Zheng, Genfa Zhu, Fengxi Yang

**Affiliations:** Guangdong Key Laboratory of Ornamental Plant Germplasm Innovation and Utilization, Environmental Horticulture Research Institute, Guangdong Academy of Agricultural Sciences, Guangzhou, 510640 People’s Republic of China

**Keywords:** *Arundina graminifolia*, Flowering, Hormone signaling, WGCNA, Transcriptome

## Abstract

**Background:**

Manipulation of flowering time and frequency of blooming is key to enhancing the ornamental value of orchids. *Arundina graminifolia* is a unique orchid that flowers year round, although the molecular basis of this flowering pattern remains poorly understood.

**Results:**

We compared the *A. graminifolia* transcriptome across tissue types and floral developmental stages to elucidate important genetic regulators of flowering and hormones. Clustering analyses identified modules specific to floral transition and floral morphogenesis, providing a set of candidate regulators for the floral initiation and timing. Among candidate floral homeotic genes, the expression of two *FT* genes was positively correlated with flower development. Assessment of the endogenous hormone levels and qRT-PCR analysis of 32 pathway-responsive genes supported a role for the regulatory networks in floral bud control in *A. graminifolia*. Moreover, WGCNA showed that flowering control can be delineated by modules of coexpressed genes; especially, MEgreen presented group of genes specific to flowering.

**Conclusions:**

Candidate gene selection coupled with hormonal regulators brings a robust source to understand the intricate molecular regulation of flowering in precious orchids.

**Supplementary Information:**

The online version contains supplementary material available at 10.1186/s12870-021-03350-6.

## Background

Flowering plants are the dominant component of terrestrial landscape and play a key role in human life. The time of flowering is crucial for the species to adapt in their environment as their life cycle runs depending on the fine combination of intrinsic and extrinsic stimuli. From the perspective of horticultural importance, the flower timing often affects the quality of floriculture crops. Stability of flowering time is the key goal for breeding endeavors because it guarantees the reliable production of crops. In recent decades, mining of genes regulating flowering pathways has been the major focus to induce breeding novelty in floriculture crops. Information from different pathways is synthesized through floral integrator genes, whose expression instigates the transition of apical meristem from vegetative to floral phase [1].

Environmental variations, such as changes in light, temperature, and hormone levels, control flower quality and flowering time [2]. Flowering time in *Arabidopsis* depends on five genetic pathways: photoperiod, aging, gibberellin, vernalization, and autonomous pathways [3]. These pathways are integrated by floral integrators, including *SUPPRESSOR OF OVEREXPRESSION OF CO 1* (*SOC1*), *FLOWERING LOCUS D* (*FD*), and *FLOWERING LOCUS T* (*FT*) [2–4]. These integrators trigger the floral morphogenesis program by transmitting the floral induction signals to the floral meristem identity genes, including *APETALA1* (*AP1*) and *LEAFY* (*LFY*). Then, floral organ development continues under the control of MADS-box genes and their co-regulators [4].

Orchids contain several MADS-box genes that regulate flowering and flower development. Based on their expression patterns and putative roles in floral organs, advanced models have been proposed for flower development in orchids [5, 6]. Recently, an increasing number of flowering genes have been functionally characterized in various orchid species, including homologs of *FT* and the gene that encodes its interacting protein, *FD*, in *Phalaenopsis aphrodite, Oncidium* Gower Ramsey, and *Dendrobium nobile*; *CONSTANS-like genes* and *LEAFY* in *Phalaenopsis aphrodite*; and genes for co-regulated transcription factors such as *CINCINNATA-like* (*TCP-like*) and *SQUAMOSA promoter binding-like genes* (*SPL-like*) [7–12].

Some of these homologs exhibit conserved functions in orchids. *PaFT1* (*Phalaenopsis aphrodite FLOWERING LOCUS T1*) is upregulated at low temperatures and induces early flowering in rice (*Oryza sativa*) and *Arabidopsis* [13]. PaFT1-interacting protein (PaFD) partially complements the late-flowering phenotype of the *Arabidopsis* mutant *fd-3* [13]. *OnFT* (*Oncidium* Gower Ramsey *FLOWERING LOCUS T*) and *TFL1* (*TERMINAL FLOWER 1*) encode the orchid homologs of the floral activator FT and the repressor TFL1, respectively [7]. The balance between these homologous proteins (FT and TFL1) controls the determinate and indeterminate growth in plants and also modulates plant architecture, thereby regulating the vegetative and reproductive organ pattern from apical meristem [14, 15]. *Dendrobium nobile MOTHER OF FT* (*DnMFT*) and *FLOWERING LOCUS T* (*DnFT*) show opposite expressions against change in temperature. Together, these studies inform our understanding of the relationships between flower development and floral regulatory genes in orchids [16].

Phytohormones play important roles in orchid flowering [17]. Auxin acts as a morphogen [18–22] and provides cues for tissue specification in a concentration-dependent manner [23]. Application of a synthetic cytokinin, 6-benzylaminopurine (BA), promoted flowering in *Phalaenopsis* and *Dendrobium* orchids, but auxin counteracted this effect. The effect of BA on flowering is enhanced when applied in combination with gibberellic acid (GA_3_) [24]. GAs regulate important developmental processes like flowering time [25, 26] and stem elongation [27–29]. Abscisic acid (ABA) is a pivotal factor that regulates bud break and flowering time [30, 31]. Strigolactones also play important roles through their cross-talk with other hormones, such as auxin, cytokinin, GA, and ethylene [32–34]. Despite their potential for wide use, the molecular underpinnings of the effects of phytohormones on orchid flowering remain unclear.

Orchidaceae is one of the largest angiosperm families and contains ornamental orchids [35, 36]. Because of their horticultural importance, more than 100,000 orchid species have been cultivated throughout the world. Most commercially popular orchid cultivars, such as *Phalaenopsis*, *Dendrobium*, and *Cymbidium*, bloom in specific seasons, under low temperature of about 5–10 °C [37]. Unlike these orchids, *Arundina graminifolia* blooms year round, peaking between September and January. It is commonly known as the “Bamboo Orchid” and is found in tropical and sub-tropical regions in Asia [38–41]. It grows on grassy slopes, under shrubs, along ravines, or in forest areas, at altitudes between 400 and 2800 m, showing a strong adaptability and a high rate of flowering and fruiting throughout the year [41]. Due to its beautiful flowers and extended flowering time, *A. graminifolia* is widely grown in Singapore, Malaysia, among other places, as a landscape and potted plant. *A. graminifolia* is used as a medicinal plant in China because of the presence of flavonoids, stilbenoid, and phenols in its extracts, which exhibit antioxidant, anti-virus, anti-tumor, and other medicinal properties [42]. Researchers have so far focused on the identification of the chemical components, pharmacological activities, and bioactive substances in *A. graminifolia*. However, the underlying molecular regulation is not discussed.

Here, we report the first de novo transcriptomic analysis of *A. graminifolia*. We compared the transcriptomic profiles at different stages of flower development and those of vegetative tissues. We elucidated the transcriptome dynamics and the gene regulatory networks associated with flower development. We identified highly expressed gene modules, unique flower-specific markers, circadian clock integrators, and hormonal regulators in different flower development stages, and in mature flowers, capsules, leaves, and roots. Our study, thus, provides valuable insights into the molecular regulatory network that regulates flowering in *A. graminifolia*.

## Results

### Perpetual flowering and flower ontogeny of *A. graminifolia*

*A. graminifolia* achieves reproductive maturity 6 months after transplantation from a culture medium. This is much shorter than the two- to three-year long juvenile phase of other orchids, such as *Phalaenopsis, Oncidium*, *Dendrobium,* and *Cymbidium. A. graminifolia* blooms all year round, with the most vigorous flowering from September to January (Supplementary Fig. 1). Its inflorescence is a raceme with an average of 6.1 flowers per plant. The average life span of a single flower is 32.3 days. The ornamental period of the whole inflorescence may span up to 5 months (Table 1).

**Table 1:** Morphology of reproductive growth of *A. graminifolia*

Time after first blooming (month)	Number of leaves	Plant height (cm)	Stem diameter (mm)	Pedicel			
				Section number	Diameter (mm)	Length (cm)	Total length (cm)
1	17.05 ± 0.64^a^	34.05 ± 1.51^a^	4.23 ± 0.11^a^	2.00 ± 0.00^a^	0.85 ± 0.03^a^	1.62 ± 0.20^b^	3.23 ± 0.40^b^
2	15.85 ± 0.55^b^	34.48 ± 1.48^b^	3.83 ± 3.76^b^	3.05 ± 0.17^b^	1.69 ± 0.04 ^b^	2.82 ± 0.16^c^	6.90 ± 0.70^a^
3	15.45 ± 0.60^b^	35.57 ± 1.39^c^	3.59 ± 0.08^c^	4.50 ± 0.48^c^	1.64 ± 0.04 ^b^	1.62 ± 0.08^b^	12.61 ± 1.17^c^
4	14.13 ± 0.60^c^	36.03 ± 1.47^d^	3.13 ± 0.08^d^	5.73 ± 0.40^d^	1.40 ± 0.05 ^c^	1.39 ± 0.09^a^	15.00 ± 1.29^d^
5	12.75 ± 0.63^d^	36.21 ± 1.48^d^	3.07 ± 0.08^d^	6.45 ± 0.50^e^	1.40 ± 0.09 ^c^	1.38 ± 0.18^a^	15.22 ± 1.35^e^

The symbols (a, b, c, d, e) show statistically significant difference at *p* < 0.05

**Fig. 1:**
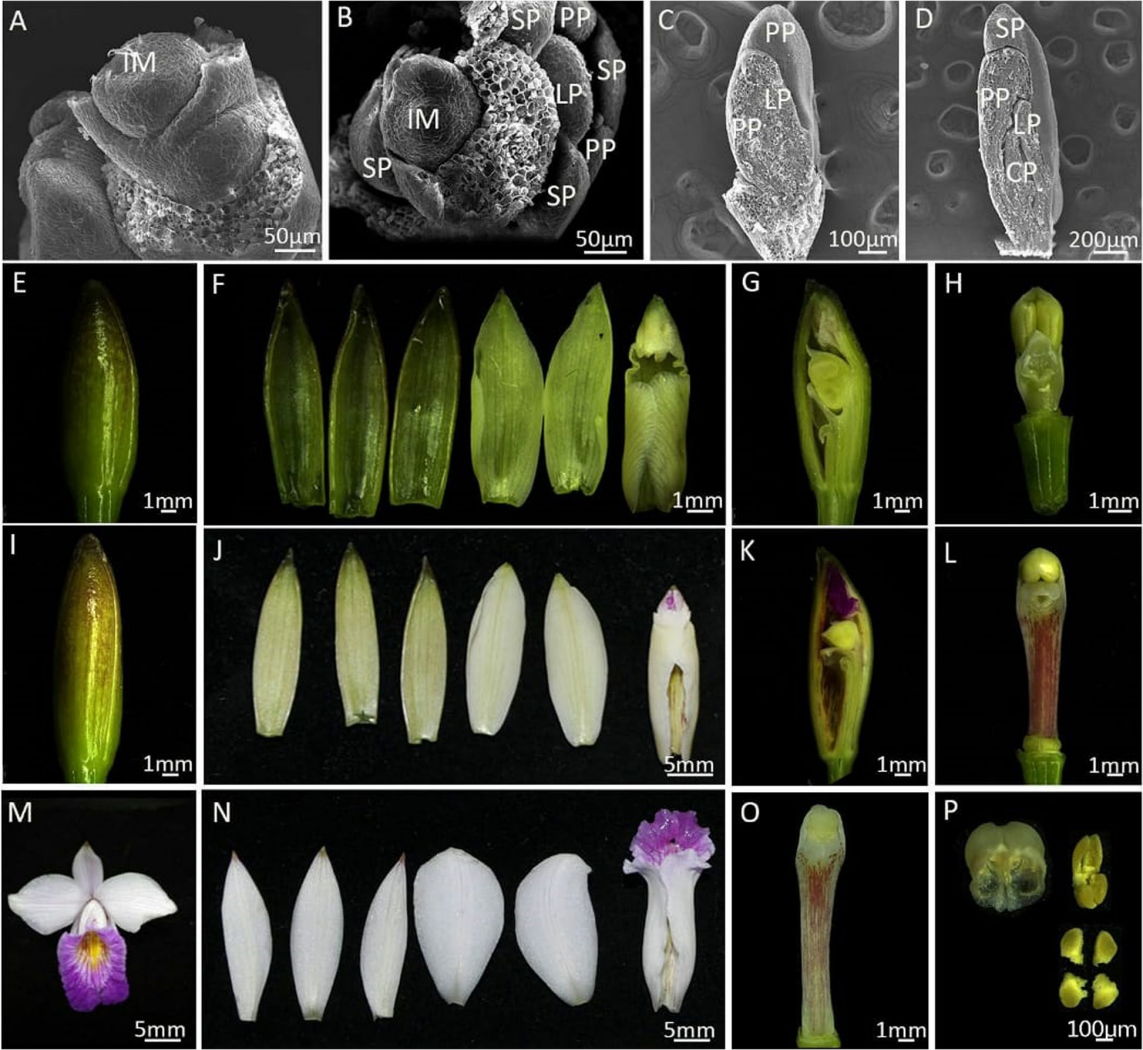
Stages of flower development in *Arundina graminifolia*. **A-D**: Scanning electron micrograph (SEM) of early floral developmental stages. **A**: Stage 0 (IM: inflorescence meristem); **B-C**: Stage 1 (IM: inflorescence meristem, PP: petal primordia, SP: sepal primordia); D: Stage 2 (PP: petal primordia, SP: sepal primordia, LP: lip primordia, CP: carpel primordia); E-H: Stage 3; I-L: Stage 4; M-P: Stage 5; P: mature flower

We observed the morphogenesis and surface structure of the floral organs under a scanning electron microscope (Fig. 1). Flower development takes place in six stages (stages 0–5). In stage 0, the inflorescence meristem (IM) is formed and has a flattened and oval primordium (Fig. 1a). In stage 1, the IM displays a floral structure on the edge of the shoot, and the floral primordia divide into sepal, petal, labellum, and column primordia. Even though the process is similar to that of other orchid species, the pace of development is much faster, with a completion time of 2–3 days in *A. graminifolia*. The floral organs are undifferentiated at this stage, and the base is connected (Fig. 1b,c). In stage 2, the floral organs differentiate continuously and establish the zygomorphy typical of orchid flowers (Fig. 1d). It takes about 10 days to grow into stage 3. In this stage, the floral apex resembles an inverted triangle (Fig. 1e): the outer sepals overlap with the inner petals (Fig. 1f and g), and the labellum develops a crooked to undulated margin (Fig. 1h). Stage 4 commences after 1 week, wherein the gynostemium elongates and the labellum acquires its coloring (Fig. 1i). Then the pollinia mature and the flower opens within 3 days (stage 5, Fig. 1m-p). The mature flower has a fine-structured column in the center (Fig. 1o), with four pollinia on a semi-circular viscidium (Fig. 1p).

### Transcriptome sequencing and functional annotation

We produced a total of 71.2 billion high-quality reads from. Each sample produced 10.8–12.8 Gb of data and an average of ~ 7.8 billion reads (Supplementary Table 1). We filtered these into 94,317 transcripts and 25,353 unigenes (Supplementary Table 2), with mean lengths of 1291 bp and 1198 bp, respectively. We annotated them using Gene Ontology (GO), Kyoto Encyclopedia of Genes and Genomes (KEGG), database of protein families (Pfam), SwissProt, and evolutionary genealogy of genes: Non-supervised Orthologous Groups (eggNOG) and non-redundant (NR) protein databases (Table 2). We identified a total of 17,171 (67.73%) unigenes with GO annotations for biological processes, molecular functions, or cellular components. We annotated 9234 genes into KEGG categories. We obtained the maximum number of annotations using eggNOG, where 18,854 (74.37%) reads were assigned to different categories, followed by NR database in which 72.29% of the reads were annotated to non-redundant proteins.

**Table 2:** Annotation statistics of DEGs for different databases

DB	Num	Ratio (%)
All	25,353	100.00
GO	17,171	67.73
KEGG	9234	36.42
Pfam	16,271	64.18
SwissProt	14,334	56.54
eggNOG	18,854	74.37
NR	18,327	72.29

Signal transduction, posttranslational modification, transcription, carbohydrate transport and metabolism, intracellular trafficking and amino acid transport, and metabolism were the major functional categories in the transcriptomic data annotated by eggNOG (Supplementary Fig. 2a). In GO annotations, a significant number of genes were annotated to transcriptional regulation, transcription, and protein phosphorylation. Most of the genes were localized to nucleus, cytoplasm, membranes, and chloroplast. Protein binding was the most prominent molecular function across all the annotated genes (Supplementary Fig. 2b). Most KEGG-annotated genes were related to translation, carbohydrate metabolism, and lipid and energy metabolism (Supplementary Fig. 2c).

*A. graminifolia* shared 7820 unigenes with orchids, such as *Phalaenopsis equestris*, *Dendrobium officinale*, and *Apostasia shenzhenica*. In pair-wise comparisons, *A. graminifolia* shared 8778, 9925, and 10,012 genes with *P. equestris*, *D. officinale*, and *A. shenzhenica*, respectively (Supplementary Fig. 2d). Only 64 genes were unique to *A. graminifolia*, compared with 615 in *P. equestris*, 1132 in *D. officinale*, and 615 in *A. shenzhenica*. The GO analysis resulted in 136 annotated terms for genes unique to *A. graminifolia*; those involved in biological process (80), metabolic process (34), and response to stimulus (27) were relatively over-represented. However, only 17 terms were annotated to cellular components, and 1 to the molecular function category.

### Identification of Transcription Factor (TF) families

Our data contained 687 TFs belonging to 35 different families (Supplementary Fig. 4). Famous TF families were found in the transcriptome, including bHLH, WRKY, MYB, CYC, bZIP, TCP, and MAD. The most abundant TF family was bHLH (70 members), followed by MYB (50 members) and WRKY (45 members). The bHLHs and MYBs were expressed significantly in the root, leaf, and early FD stages. However, WRKYs were mainly expressed in the root and flower (Supplementary Fig. 3).

Tissue specific up- and downregulation modules were found for TF families (Supplementary Fig. 5). Interesting variations can be found among TFs families for stages of flower development or tissue types (Supplementary Fig. 4a). The most upregulated TFs (435) were found in FD1, whereas the most downregulated TFs (495) were found in FD5 (Supplementary Fig. 5b). An equal ratio of up- and downregulated TFs can be seen in the mature flower (Supplementary Fig. 4c).

### Differential expression of flowering-related genes

**Fig. 2:**
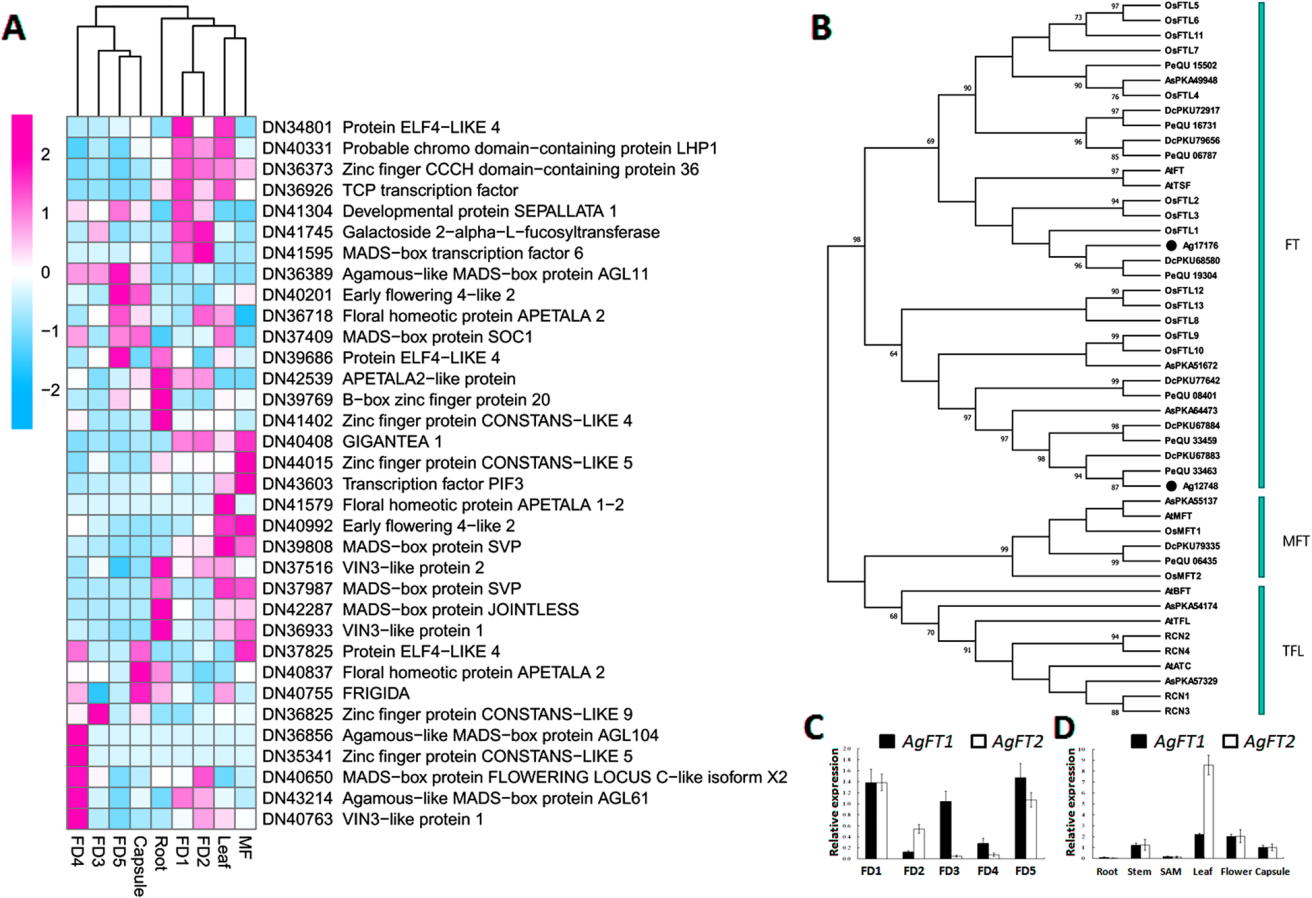
DEGs relating to flowering control. **A**: heatmap of flowering-related DEGs; **B**: Phylogenetic tree of *AgFT* genes with FT, MFT, and TFL genes from other species; **C**: qRT-PCR expression of *AgFT1* and *AgFT2* in flower development stages; **D**: qRT-PCR analysis of *AgFT1* and *AgFT2* in tissues other than flower

We identified 36 floral-related DEGs (Fig. 2a). Genes encoding homologs of the floral regulators, *SEP*, *ELF*, *MADS*, *TCP*, *AGL*, *FT,* and *LHP*, were expressed in FD1 (Fig. 2a). *ELF* works in the photoperiodic pathway (GO: 0009648) and controls rhythmic process (GO: 0048511) of organ development. TCP21 is a transcription factor that regulates the circadian rhythm (GO: 0042752).

Two isoforms of *MADS6* and *FT* showed high expression in FD2. *MADS6* is involved in the specification of floral organ identity and meristem determinacy (K03217). An isoform of *COL9* was the only CONSTANS-like zinc finger protein expressed in FD3. It regulates circadian rhythms (GO: 0007623) and flowering (GO: 0048579).

AGAMOUS-like (AGL) MADS-box proteins (AGL104, AGL61, and AGL65) were expressed in FD4, involving pollen development (Fig. 2a). Moreover, COL (CONSTANS-like zinc finger protein) was also expressed in FD4 and it controls regulation of flower development (KO3539). In stage 5 of flower development, *AGL11* and *EFLs* were upregulated. *EFL3* and *4* are floral repressors that coordinate floral transition through the photoperiod pathway.

Two types of *COs*, two *EFL* types, *GI*, and *PIF3* were expressed in mature flower (Fig. 2a). *GIGANTEA* (*GI*) is a protein that regulates the circadian clock (K12124). We observed a high level of *GI* expression in mature flower.

### Validation of *AgFT* genes correlated with reproductive development

Flowering Locus T (FT) family genes function as key flowering integrators: FT promotes flowering and TFL1 inhibits flowering. To comprehensively identify the FT genes in *A. graminifolia*, we used the *Arabidopsis* and rice FT amino acid sequences to screen for homologs in our dataset using tBLASTN. FT gene number varied among species (Fig. 2b). There are 6, 7, and 8 FT-like genes in *Apostasia*, *Phalaenopsis*, and *Dendrobium*, respectively. However, *A. graminifolia* contained only 2 FT-like genes, and no TFL1-like or MFT-like genes.

*AgFT* genes showed the lowest expression levels in root and shoot apical meristem (SAM), and high levels in leaf and flowers, followed by stem and capsule (Fig. 2d). This pattern is similar to the FT expression pattern in other species, and is associated with floral bud initiation. Both *AgFT1* and *AgFT2* were highly expressed in floral developmental stages 1 and 5, but had lower expression in stages 2–4 (Fig. 2c). Moreover, we observed a complementary expression pattern of *AgFT1* and *AgFT2* during stages 2–4: *AgFT1* showed higher expression in stage 2, while *AgFT2* showed higher expression in stages 3 and 4.

### Hormonal regulation possesses a fundamental role in flower development

**Fig. 3:**
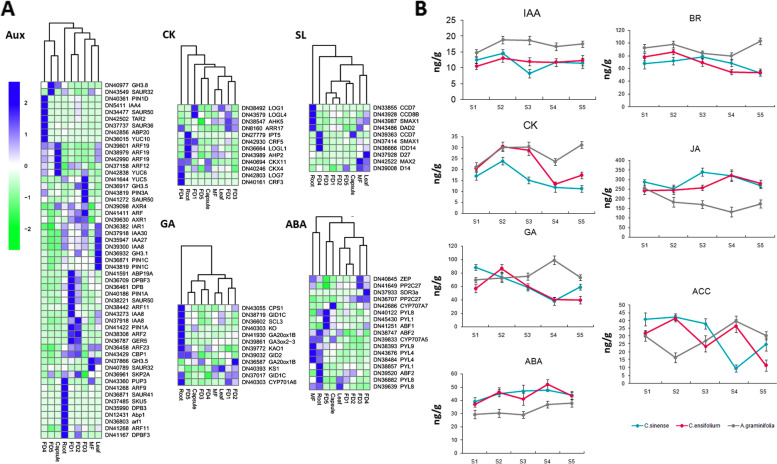
**A**: DEGs involved in auxin biosynthesis and signaling, cytokinins biosynthesis and signaling, strigolactone biosynthesis and signaling, gibberellin biosynthesis and signaling, and ABA signaling and biosynthesis; **B**: Comparison of hormone levels in *A. graminifolia*, *C*. *ensifolium* and *C. sinense*

We mined a number of genes involved in the biosynthesis and signalling of auxins (Fig. 3a). Notably, *TAR2*, a candidate gene involved in auxin biosynthesis (GO: 0009851), was expressed only in FD4. *SKP2A*, responsible for auxin-activated signaling (GO: 0009734), was expressed in FD2 (Fig. 3a). Auxin efflux carriers (*PINs*) were expressed mainly in FD4, and *ARFs* showed high expression in the capsule or early stages of flower development.

Cytokinins interact with auxins to control organogenesis. We observed higher expression of cytokinin-related genes in FD4 than in other stages of flower development, or in other tissue types (Fig. 3a). *LOGs* are involved in cytokinin biosynthesis (GO: 0009691). *LOG7* was expressed in FD4. *LOG1* appeared in three isoforms, in leaf, FD5, and FD1. *CRF3*, which acts in the cytokinin-activated signaling pathway (GO: 0009736), was expressed only in FD4. *CRF5* exhibited low expression in FD4 and elevated expression in root and FD1.

We also identified genes involved in strigolactone biosynthesis and signaling (Fig. 3a). These genes were not expressed in the first three stages of flower development. The highest expression levels were in FD4 and roots. *D27* is responsible for strigolactone biosynthesis (GO: 1901601) and was only expressed in leaf. *CCD7*, another strigolactone biosynthesis gene (GO: 1901601), showed highest expression levels (FPKM: 164.07) in FD4. *SMAX1*, a transcriptional corepressor that confers to response to strigolactone (GO: 1902347), also displayed high expression levels in FD4 (Fig. 3a).

We identified 72 annotated genes related to gibberellin. A heatmap of these annotations showed that most of the genes had high expression levels in root, followed by FD1 and FD2 (Supplementary Fig. 5). Two *DELLA* genes (*GAI*, *SLR-like*) were repressed along flower development (Fig. 3a). Reduced expression of *DELLA* genes promotes floral induction and development.

Abscisic acid is a general negative regulator of plant organ development, and interacts with other hormones to control flowering. We identified 19 transcripts related to ABA biosynthesis, metabolic process, ABA-activated signaling, and ABA binding. Most of these genes showed significant expression intensities in roots (Fig. 3a).

### Correlation between the endogenous hormones and flower development

To validate the effect of hormones on flower development, we investigated the levels of endogenous hormones—cytokinins (CK), gibberellic acid (GA), abscisic acid (ABA), brassinosteroid (BR), jasmonic acid (JA), 1-Aminocyclopropane-1-carboxylic acid (ACC), and indoleacetic acid (IAA)—at different growth stages (Fig. 3b). These were then compared with the corresponding hormone levels in the seasonal-flowering orchid species, *Cymbidium sinense* (spring) and *Cymbidium ensifolium* (summer).

All hormones except ACC varied significantly among these orchids. *A. graminifolia* had 1.3- to 2.5-fold higher levels of GA3, CK, IAA, and BR than the seasonal-flowering orchids. The levels of these hormones increased during floral bud development: FD5 buds had 13–50% more hormones than FD1 buds. In a reversal of this pattern, FD5 buds of *C. sinense* and *C. ensifolium* had 20–40% lower levels of these hormones than FD1 buds. *A. graminifolia* had considerably lower levels of JA and ABA—which are negative regulators of floral organ development—than *C. sinense* and *C. ensifolium* (Fig. 3b).

We validated the hormone responsive genes in the GA and ABA pathways by qRT-PCR. This analysis included five GA-related genes (*GID1*, *GA20OX*, *GA3OX*, *GAI*, and *SLR*) and four ABA-related genes (*ABF1*, *ABF2*, *PYL4*, and *CYP707A5*; Fig. 5b). *GA3OX* was expressed predominantly in the root, and *GID1* and *GA20OX* were expressed to comparable levels in all plant parts*.* The DELLA genes (*GAI* and *SLR*) showed decreasing expression levels during bud development, corroborating the DEG analysis. *ABF1* had higher expression levels in FD1 than in other stages or tissues. *ABF2* showed high expression in FD1 and FD2. *CYP707A5* was expressed mainly in roots, and its levels decreased during floral bud development. *PYL4* was expressed mainly in leaf and root.

### Identification of coexpressed gene modules for flowering

**Fig. 4:**
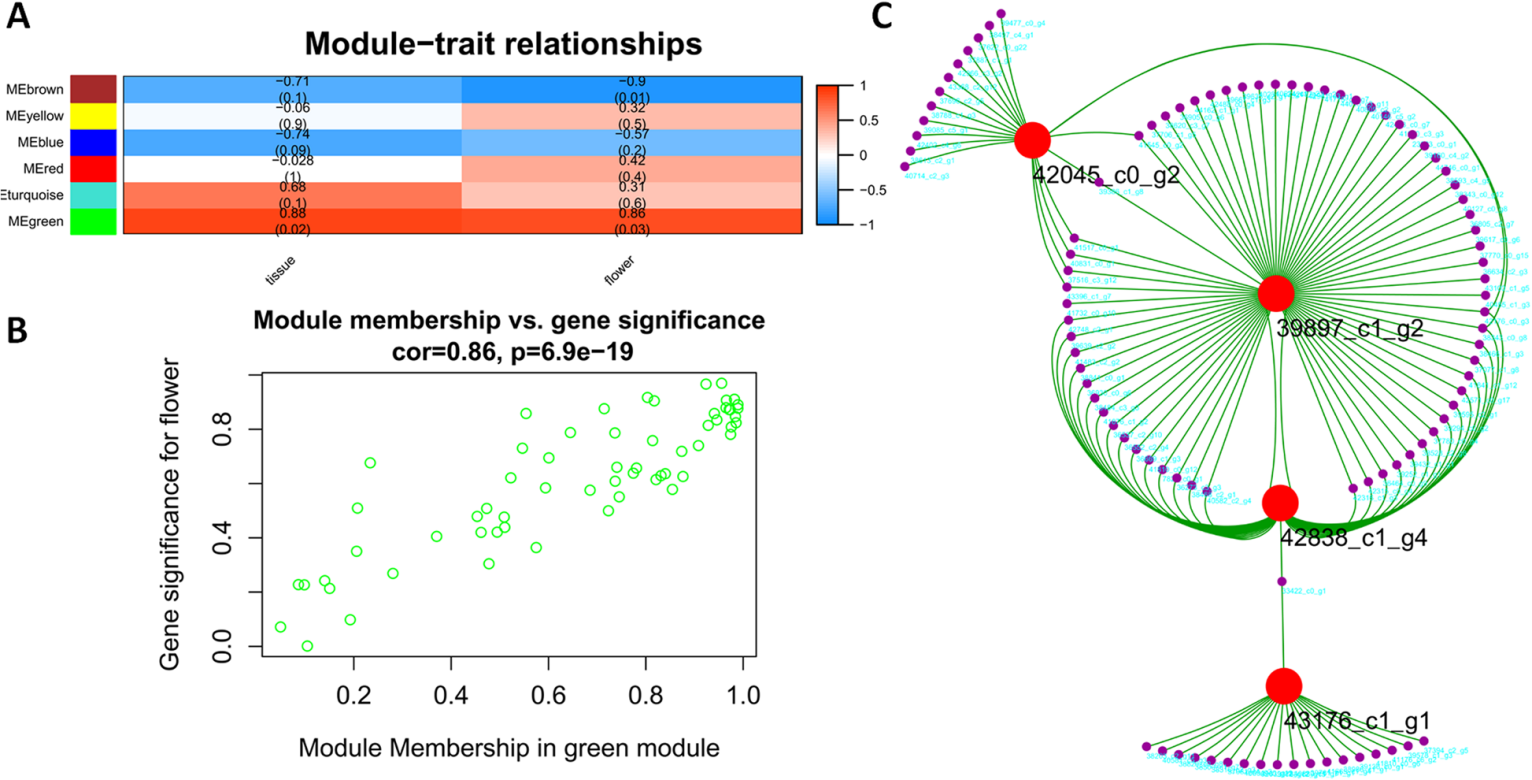
Weighted Gene Coexpression Network Analysis (WGCAN). **A**: Gene modules related to flowering and tissue types; **B**: Gene significance to flowering; **C**: Identification of four hub genes from green module

To better understand the gene regulatory networks during flower development, we performed WGCNA in association with flowering and tissues (Fig. 4). Six modules were observed for flowering. MEgreen was the most prominent for flowering (Fig. 4a). A cluster of genes can be seen with a high correlation value 0.86 in MEgreen module (Fig. 4b). From this module, some hub genes were identified using Cytoscape with their intra- and inter-module interactions (Fig. 4c).

The 39897_c1_g2 is annotated as DRE-binding protein DREB1. It is an ethylene-responsive transcription factor (RAP2–13) with GO annotation as ethylene-activated signaling pathway (GO: 0009873). 43176_c1_g1 is a bibenzyl synthase (BIBSY212), involving various biological processes, such as response to auxin (GO: 0009733), response to jasmonic acid (GO: 0009753), flavonoid biosynthetic process (GO: 0009813) and auxin polar transport (GO: 0009926). 42045_c0_g2 is annotated as protein ECERIFERUM (CER1) with biological process of anther development (GO: 0048653). 42838_c1_g4 is predicted to be homeobox-leucine zipper protein ATHB-5 and may involve ABA regulation, including response to ABA (GO: 0009737), ABA-activated signaling pathway (GO: 0009738) and regulation of ABA-activated signaling pathway.

### The qRT-PCR validation of selected DEGs

**Fig. 5:**
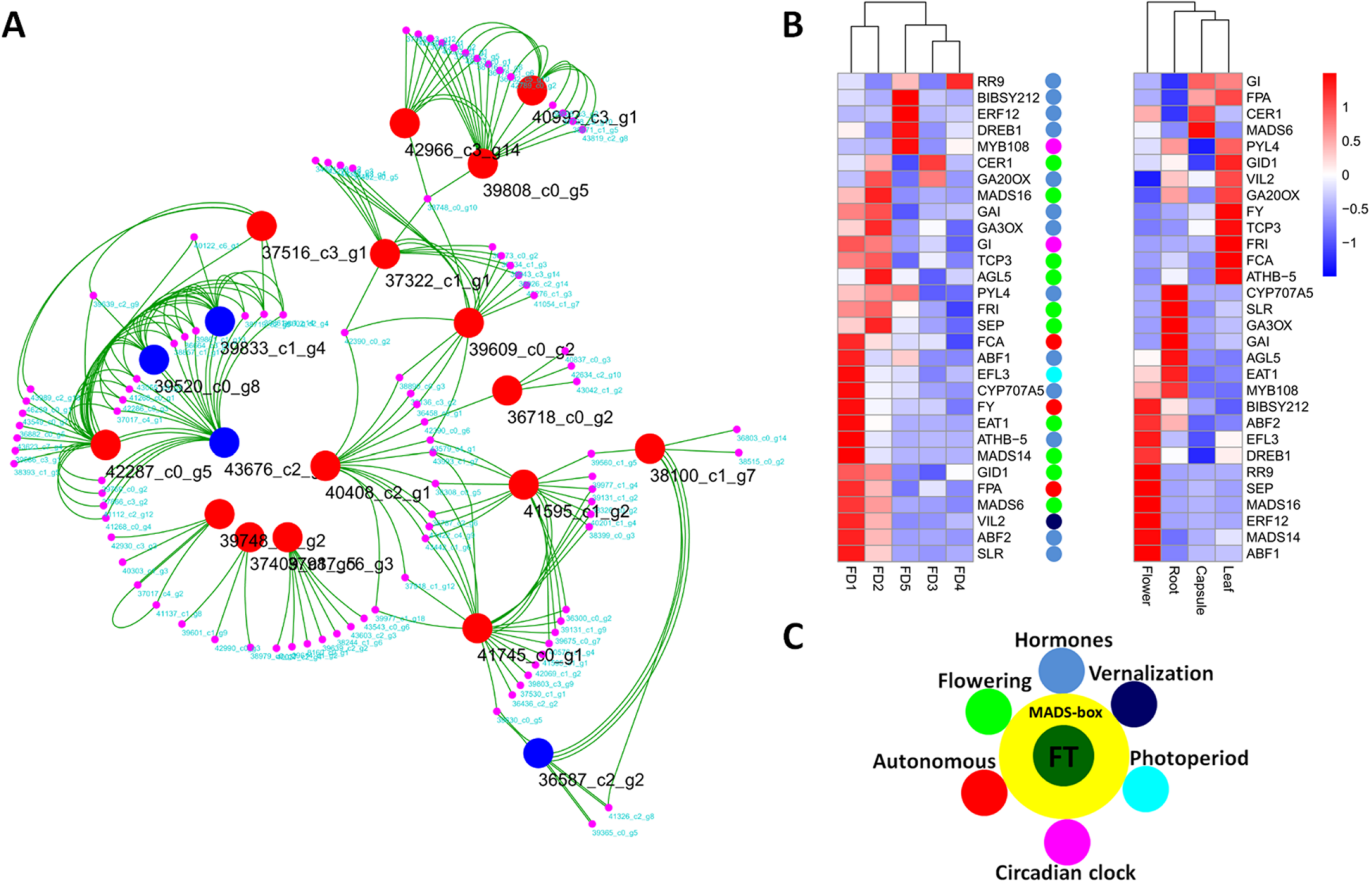
Hub genes from coexpressed modules related to flowering and hormonal regulation (**A**) (blue circles show the key hormonal regulators and the red circles show floral regulators), qRT-PCR analyses of 30 selected genes related to multiple pathways (**B**) and the hypothetical model of flowering regulation by different pathways (**C**)

All the DEGs related to flowering and hormonal regulation were sorted and coexpressed modules were generated. This identified a number of hub genes that play important roles in flowering (Fig. 5a). A total of 32 DEGs were quantified through qRT-PCR to ascertain their roles in flower development (Fig. 5b). The qRT-PCR expressions of most of the DEGs were consistent with that of transcriptomic expressions. Clustering of qRT-PCR expressions showed that most of the DEGs were expressed in FD1 among the stages of flower development, representing different pathways of flowering regulation. Most of the qRT-PCR detected DEGs showed similarities to the genes identified in other orchids, such as *Cymbidium sinense*, *Cymbidium goeringii*, *Dendrobium catenatum*, and *Phalaenopsis equestris* (Supplementary Table 4). Moreover, a hypothetical model can be proposed based on the variety of regulatory genes found in our data (Fig. 5c).

## Discussion

*Arundina graminifolia* is unique among orchids due to continuous flowering, but the molecular regulation of this flowering pattern remains poorly understood. We used de novo RNA-seq to study the transcriptome dynamics of flower development. Several genes were expressed during flower development stages and in various tissue types. We identified homologs of key flowering regulators, such as *ELF*, *COL4*, *COL5*, *COL9*, FT, and SOC1, in *A. graminifolia*. Homologs of *GA20ox1B*, *GID1C*, *GID2*, *GA3ox2*, *GAI*, and *SLR* in the GA pathway, ABA pathway genes (ABF1, ABF2 and PYL4) and homologs of *FCA*, *FPA*, and *FY* in the autonomous pathway were identified in this study. Important transcription factor families were also rich in the data. Contents of seven hormones were quantified in *A. graminifolia* and compared with *C. sinense* and *C. ensifolium*. Therefore, our data from *A. graminifolia* captures genes from classical flowering pathways that have been identified in *Arabidopsis* [3].

Our data suggested important TFs that expressed to specific stages of flower development (Fig. 5b). MADS14 is an AP1 (APETALA1)/FUL (FRUITFUL)-like MADS-box TF that regulates floral meristem identity [43–45]. It showed the highest expression in FD1 as compared to other stages of FD (Fig. 5b). MADS6, MADS16 and SEP showed high expressions in FD1 and FD2. MYB108 along with MYB24 regulates JA-mediated stamen maturation in *Arabidopsis* [46]. It was highly expressed in FD5. EAT1 (EXTERNAL TAPETUM1) is a bHLH TF involving tapetal cell-fate decision [47] and showed high expression in FD1. RR9 (type-B response regulator) showed high expression in FD4 and FD5. ERF12 is a transcriptional repressor in the photoperiod pathway and regulates floral timing and floral organ identity along with AP2 (APETALA2) [48, 49]. It was mainly expressed in FD5 (Fig. 5b).

The transcriptome of *A. graminifolia* contained two *FT* homologs, but no *TFL1* homologs. *FT* genes provide important sources of flowering-time variation in different plant species or cultivars. *FT* is regulated antagonistically by the photoperiod and vernalization pathways and plays a central role in integrating flowering [50]. Sequence comparisons clustered the FT homologs into three major clades: the *FT*, *MFT*, and *TFL1* subfamilies. FT and MFT function as flowering promoters, whereas TFL1 is associated with vegetative development and maintenance of meristem indeterminacy. Seasonal orchid species contain comparable numbers of flowering-related MADS-box. The contraction of the FT gene family members may be a unique characteristic of this species.

Bud growth is promoted by endogenous hormones such as GA3, CK, IAA, and BR, and is inhibited by ABA, JA, and ACC [51–56]. We quantified the hormonal contents in *A. graminifolia* compared with the typical seasonal flowering species (*C. sinense* and *C. ensifolium*). The levels of GA3, CK, IAA, and BR in *A. graminifolia* are significantly higher than those in seasonal-flowering orchids. In line with our expectations, the levels of the inhibitory factors are higher in seasonal-flowering orchids. A considerable proportion of our transcriptome data included genes that function in hormone signaling. We identified auxin homeostatic and signaling gene families, including ARFs, GH3, IAAs, PINs, SAURs and YUCs; PINs were expressed specifically at the early stages of floral development. We also detected several genes related to gibberellins in the first two stages of flower development (Supplementary Fig. 5). The expression of gibberellin biosynthesis genes (GA3OX and GA20OX) and GA signaling DELLA genes (GAI and SLR1) was high in the early stages of flower development (Fig. 5). Transcriptomic analysis shows elevated expression of FT/FD and GA biosynthesis-related genes, which promote growth, and the downregulation of ABA pathway genes at the same time may transiently release dormancy to ease bud break [57–59]. However, ABA may defy this effect in two ways: it may downregulate FT/FD at low temperatures and regulate GA levels by inhibiting SVP during short-days.

SVP (SHORT VEGETATIVE PHASE) is a flowering-time regulator. It positively regulates TCP18 [TEOSINTE BRANCHED, CYCLOIDEA, PCR (TCP)], which is a mediator of temperature-dependent bud break [60]. SVP and TCP function in a temperature-sensitive transcriptional module and mediate bud break. Moreover, SVP targets the ABA and GA pathway genes during bud break [61]. SVP also mediates photoperiodic control of dormancy, downstream of the ABA pathway, in hybrid aspen. Downregulation of SVP suppresses dormancy, whereas overexpression alleviates the dormancy defects caused by ABA insensitivity [60]. We identified two homologs of *Arabidopsis thaliana* SVP, showing the highest expression in leaves (Fig. 5a). Among the TCP homologs identified, TCP3 showed the highest expression in FD1-FD3 and TCP21 was expressed in FD1 and leaves.

Taken together, we present that multiple regulatory pathways may control flowering regulation in *A. graminifolia* (Fig. 5c). We found candidate genes from multiple regulatory conduits: photoperiod, circadian clock, vernalization, hormonal, and autonomous pathways. These pathways interact with floral integrators to regulate flowering time. FT1/FD may act as a central receiver of signals along with APETALA (AP1, AP2 and AP3) and SOC1. Moreover, ABA may control bud break through SVP at low temperature during short days. However, extended research is required to reveal these hypotheses in *A. graminifolia*.

## Conclusions

Our results show that different pathways coordinate the early and late stages of flower development. We found key regulatory genes of flowering time for different pathways, such as photoperiod (EFL3, COLs), vernalization (VIL2), circadian clock (MYB108 and GI), and autonomous (FCA, FPA, FY) pathways, and the key floral integrators of these pathways (FT, SEP, FRI, TCP3, AGL5, MADS6, and MADS16). In the hormonal pathway, we found key regulators of gibberellin (GA20OX, GA3OX, GAI, and SLR1) and abscisic acid (PYL4, ABF1, ABF2, and CYP707A5). All these genes were validated through qRT-PCR (Fig. 5). Major plant hormones were quantified in *A. graminifolia* and compared with typical seasonal flowering orchid species (*C. sinense* and *C. ensifolium*). Therefore, this mining of key regulators from different pathways sets a basis for functional characterization of these genes in orchids to decipher the complete model of flowering time regulation.

## Methods

### Plant materials and growth conditions

Plants of *Arundina graminifolia* were grown in three groups from seeds at the greenhouse of the Environmental Horticultural Research Institute of Guangdong Academy of Agricultural Sciences, China. All the groups were maintained at a day/night temperature of 25/20 °C and photoperiod of 16/8 h. Sampling was done from five floral developmental (FD) stages (stage 1–5), mature flowers, leaves, root and capsule. Nine samples were obtained for each stage of flower development or tissue type from each group. By this, each tissue was sampled 27 times from three groups. Samples were pooled and three samples were collected for RNA-sequencing, while the remaining samples were used for hormonal analyses. Thus, three technical and biological repeats were collected from each stage and tissue type. Samples were collected in liquid nitrogen and immediately stored at − 80 °C until the extraction of RNA.

### RNA-seq library preparation and sequencing

RNA was extracted from various tissue types (5 floral developmental stages, mature flower, leaf, root and capsule) using TaKaRa kit for RNA extraction. The cDNA libraries were constructed from RNA. The mRNAs were filtered from total RNA using Oligotex mRNA Midi Kit (QIAGEN, GERMANY)**.** RNA was quantified on Nano-Drop 2000 spectrophotometer (Thermo Scientific, USA) and the cDNA libraries were generated following Illumina manufacturing protocol as suggested previously [62]. In short, mRNAs were obtained from total RNA and fragments were made to an approximate length of 200 bp. Then, the isolated mRNAs were subjected to cDNA synthesis of first and second strand followed by adapter ligation and low-cycle enrichment following the instructions by TruSeq®RNA HT Sample Prep Kit (Illumina, USA). The library products thus purified were evaluated using the Agilent 2200 TapeStation and Qubit®2.0 (Life Technologies, USA), followed by dilution to 10 pM for in situ cluster generation on the HiSeq2500 pair-end flow cell and then pair end sequencing (2 × 100 bp) was performed. About 60 million reads were produced for each sample. Transcriptomic de novo assembly was performed using Trinity program with default parameters [63].

### DEG analysis

The level of gene expression was ascertained by RPKM value using the following formula:

$$\mathrm{RPKM}=\left[\mathrm{total}\ \mathrm{exon}\ \mathrm{reads}/\mathrm{mapped}\ \mathrm{reads}\ \left(\mathrm{millions}\right)\right]\ \mathrm{x}\ \mathrm{exon}\ \mathrm{length}\ \left(\mathrm{kb}\right)$$

The significant difference of gene expression among various tissue types was determined using edgeR. The false discovery rate (FDR) was used to determine the threshold *P-*value in various tests and a threshold level of significant difference in gene expression was set at FDR < 0.05 and | log2 ratio| > 1 (two fold change). Pearson correlation coefficient (PCC) was used to ascertain the correlation between different parts. Moreover, principal component analysis (PCA) and hierarchical clustering were performed using corrplot and prcomp utilities of R package [64].

The DEGs were annotated to Kyoto Encyclopedia of Genes and Genomes (KEGG) and gene ontology (GO) enrichment analyses following the method described previously [65]. Briefly, all the DEGs were first mapped to GO terms or KEGG pathways in the databases (http://www.geneontology.org/ or http://www.genome.ad.jp/), calculating gene number for each term or pathway [66, 67]. Then, the hypergeometric test was applied to find the DEGs with genetically enriched terms. A threshold *p-*value or *q*-value of ≤0.05 was set to filter significantly enriched GO terms or KEGG pathways.

### GO and pathway enrichment analyses

Clustering of selective DEGs was performed for biological process using BINGO plug-in of Cytoscape [64, 68]. For GO terms, the *P-*value was corrected following the method by Benjamini Hoschberg [64]. The GO terms with *q-*value ≤0.05 were considered as significantly enriched. Pathway enrichment analysis of different gene sets was performed using MapMan (v3.6.0RC1) with the best Arabidopsis (TAIR10) homolog as reference.

### Identification of Transcription Factor (TF) families

The term ‘transcription factor’ was searched across annotated DEGs, and identified 687 DEGs annotated as transcription factors. The TFs were sorted into 35 TF families.

### Weighted Gene Coexpression Network Analysis (WGCNA)

WGCNA was performed to identify modules related to flowering using the WGCNA packages in R, as previously reported [69].

### Real-time quantitative RT-PCR analysis

A total of 32 genes (30 hormonal and flowering pathway genes, and 2 FT homologs) were selected to check their expression trends through qRT-PCR across different plant positions. The RNA was extracted from 27 samples of 9 tissues of the plants grown in the green house of the academy as mentioned above. Total RNA was extracted from eight tissue types and reverse-transcribed to obtain cDNA following protocol by Fermentas. The cDNA was subjected to qRT-PCR in a reaction mixture volume of 20 μl containing 10 μl of SYBR premix Ex-taq™ (Takara, Japan) using Bio-Rad CFX-96 RealTime PCR System (Bio-Rad, USA). *Actin* was used as an internal standard for normalization of expression data. The experiment was performed in three biological replicates, each containing three technical replicates. The primers to clone the targeted genes were designed using Primer 5.0 software (Supplementary Table 3).

### Hormonal analysis

Hormonal analysis was performed from five stages of floral development to compare hormonal contents for *Cymbidium sinense*, *Cymbidium ensifolium* and *Arundina graminifolia*. *C. sinense* and *C. ensifolium* are typical seasonal flowering species and therefore *A. graminifolia* was compared with them to make a comparison of hormonal fluctuations between season and continuous flowering. Hormonal contents were determined following the protocol of HPLC-MS/MS (Aglient) described by Pan et al. [70].

### Scanning electron microscopy

Floral buds were collected from stage 0 to stage 2 for microscopic detailing of floral initiation. Buds were dissected and fixed in a solution containing 2% formaldehyde and 3% glutaraldehyde for 24 h. After that dehydration was done in acetone and dried at a critical-point in liquid CO_2_. Fully dried samples were fixed on stubs and sputter bearing 25 nm coating of gold. Finally, the specimens were observed under a scanning electron microscope JSM-6360LV (JEOL).

### Statistical analysis

One-way ANOVA was applied using SPSS software (SPSS Inc., Chicago, IL, USA; ver. 16.0) to analyse the hormonal and qRT-PCR data. Significant differences are shown at *p < 0.05* or *p < 0.01* level.

## Supplementary Information

**Additional file 1: Supplementary Figure 1** Plants of *A. graminifolia*. A: Whole plant; B: Opening flower; C: Mature flower. **Supplementary Figure 2.** Annotation overview. A: eggNOG functional categories; B: GO categories; C: KEGG pathway classification; D: Shared and unique proteins among *A. graminifolia*, *Apostasia shenzhenica*, *Dendrobium candidum*, and *Phalaenopsis equestris.* **Supplementary Figure 3.** Abundance of TF families; a) number of TFs in all major families, b) bHLH TF family, c) MYB TF family, d) WRKY TF family. **Supplementary Figure 4.** a) Number of up and down regulated TFs, b) stage specific number of up and down regulated TFs, c) relation of up and down regulation across different tissues. **Supplementary Figure 5.** Search results of annotation of “Gibberellins” throughout the DEGs.

**Additional file 2: Supplementary Table 1.** Flower development and plant position-based division of transcriptomic data. **Supplementary Table 2.** Total number of transcripts and unigenes identified in the transcriptome. **Supplementary Table 3.** Primers used for qRT-PCR. **Supplementary Table 4.** Similarity index of some of the important flowering related DEGs in *Arundina graminifolia* with other orchids and angiosperms.

## Data Availability

All relevant supplementary data is provided along with this manuscript as supplementary file.
