# Supplementary material for: Genetic insights into the regulatory pathways for continuous flowering in a unique orchid Arundina graminifolia
Source: BMC Plant Biol. 2021 Dec 10;21:587. doi: 10.1186/s12870-021-03350-6 (PMC8662845; doi:10.1186/s12870-021-03350-6)
Supplement: Supplementary file 1 — Additional file 1: Supplementary Figure 1 Plants of A. graminifolia. A: Whole plant; B: Opening flower; C: Mature flower. Supplementary Figure 2. Annotation overview. A: eggNOG functional categories; B: GO categories; C: KEGG pathway classification; D: Shared and unique proteins among A. graminifolia, Apostasia shenzhenica, Dendrobium candidum, and Phalaenopsis equestris. Supplementary Figure 3. Abundance of TF families; a) number of TFs in all major families, b) bHLH TF family, c) MYB TF family, d) WRKY TF family. Supplementary Figure 4. a) Number of up and down regulated TFs, b) stage specific number of up and down regulated TFs, c) relation of up and down regulation across different tissues. Supplementary Figure 5. Search results of annotation of “Gibberellins” throughout the DEGs. [file 12870_2021_3350_MOESM1_ESM.docx]

Genetic insights into the regulatory pathways for continuous flowering in a unique orchid *Arundina graminifolia*

Sagheer Ahmad ^1^, Chuqiao Lu ^1^, Jie Gao^1^, Rui Ren ^1^, Yonglu Wei^1^, Jieqiu Wu ^1^, Jianpeng Jin ^1^, Chuanyuan Zheng ^1^, Genfa Zhu^1,*^and Fengxi Yang^1,*^

1 Guangdong Key Laboratory of Ornamental Plant Germplasm Innovation and Utilization, Environmental Horticulture Research Institute, Guangdong Academy of Agricultural Sciences, Guangzhou, 510640, P.R.China; sagheerhortii@gmail.com(SA); luchuqiao@gdaas.cn (CL); gaojie@gdaas.cn (JG); Renruinjau@163.com (RR); weiyonglu@gdaas.cn (YW); Jieqiu2021520@163.com (JW); jinjianpeng@gdaas.cn (JJ); zcytrain@zhku.edu.cn (CZ); zhugenfa@gdaas.cn (GZ); yangfengxi@gdaas.cn (FY)

***** Correspondence: yangfengxi@gdaas.cn; Tel.: +86-020-8516-1014

**Supplementary Figure 1. Plants of *A. graminifolia*. A: Whole plant; B: Opening flower; C: Mature flower**

**
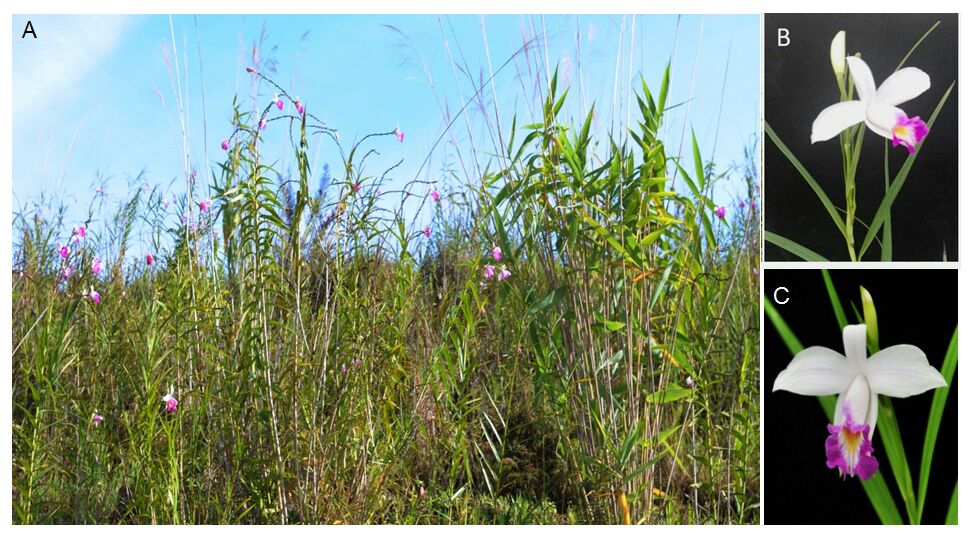
**

**Supplementary Figure 2** Annotation overview. A: eggNOG functional categories; B: GO categories; C: KEGG pathway classification; D: Shared and unique proteins among *A. graminifolia*, *Apostasia shenzhenica*, *Dendrobium candidum*, and *Phalaenopsis equestris*

**
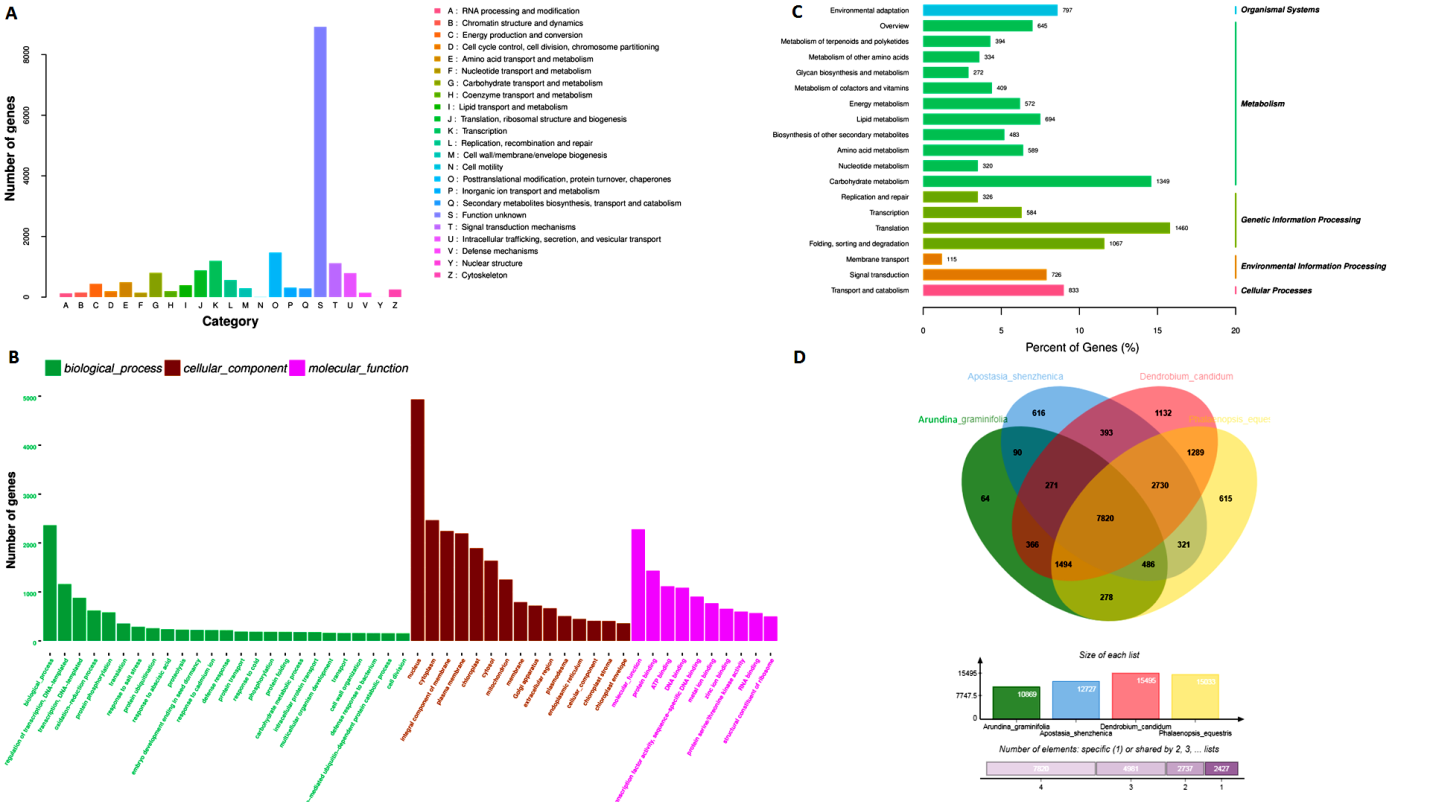
**

**Supplementary Figure 3** Abundance of TF families; a) number of TFs in all major families, b) bHLH TF family, c) MYB TF family, d) WRKY TF family

**
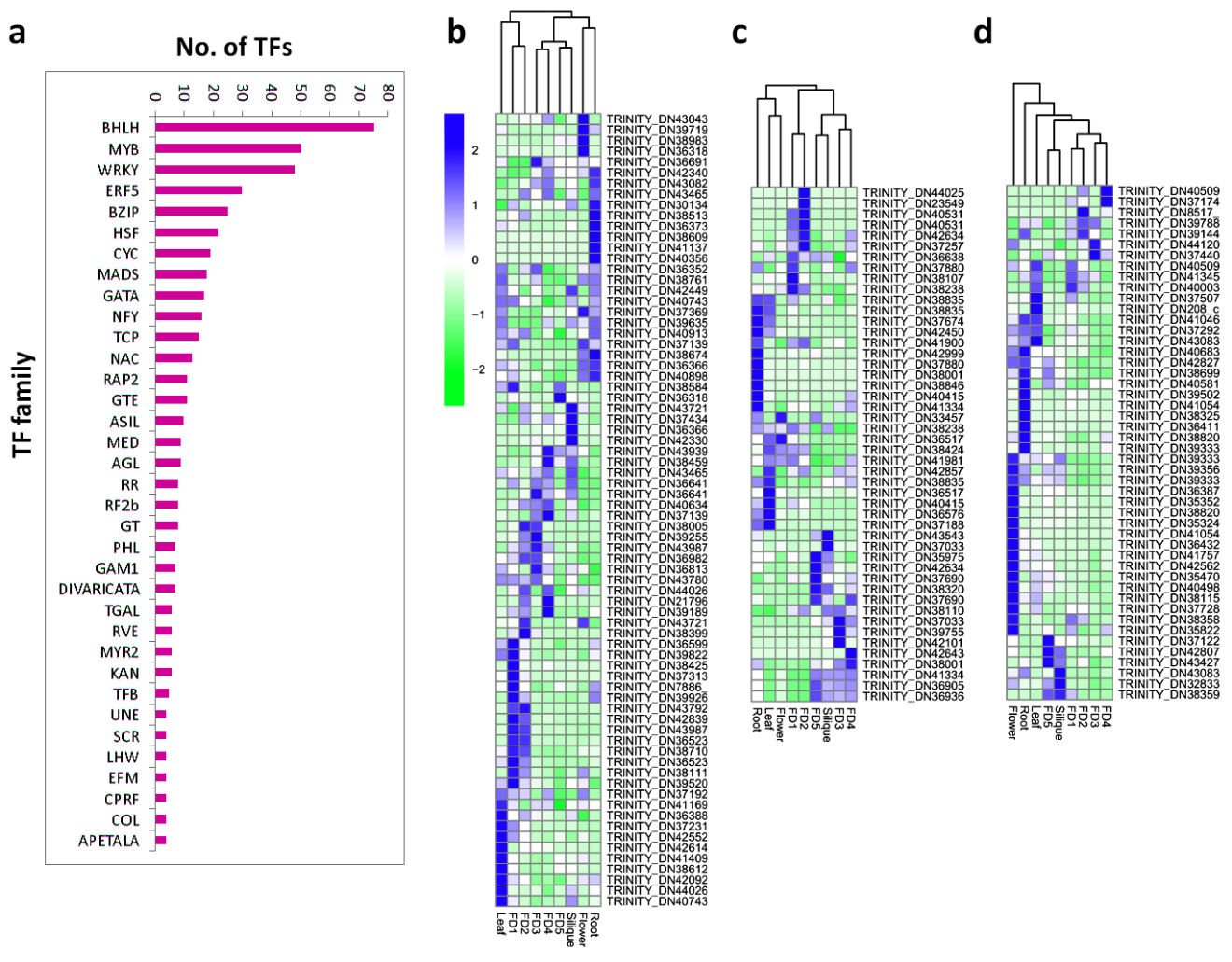
**

**Supplementary Figure 4 a)** Number of up and down regulated TFs, **b)** stage specific number of up and down regulated TFs, **c)** relation of up and down regulation across different tissues.

**
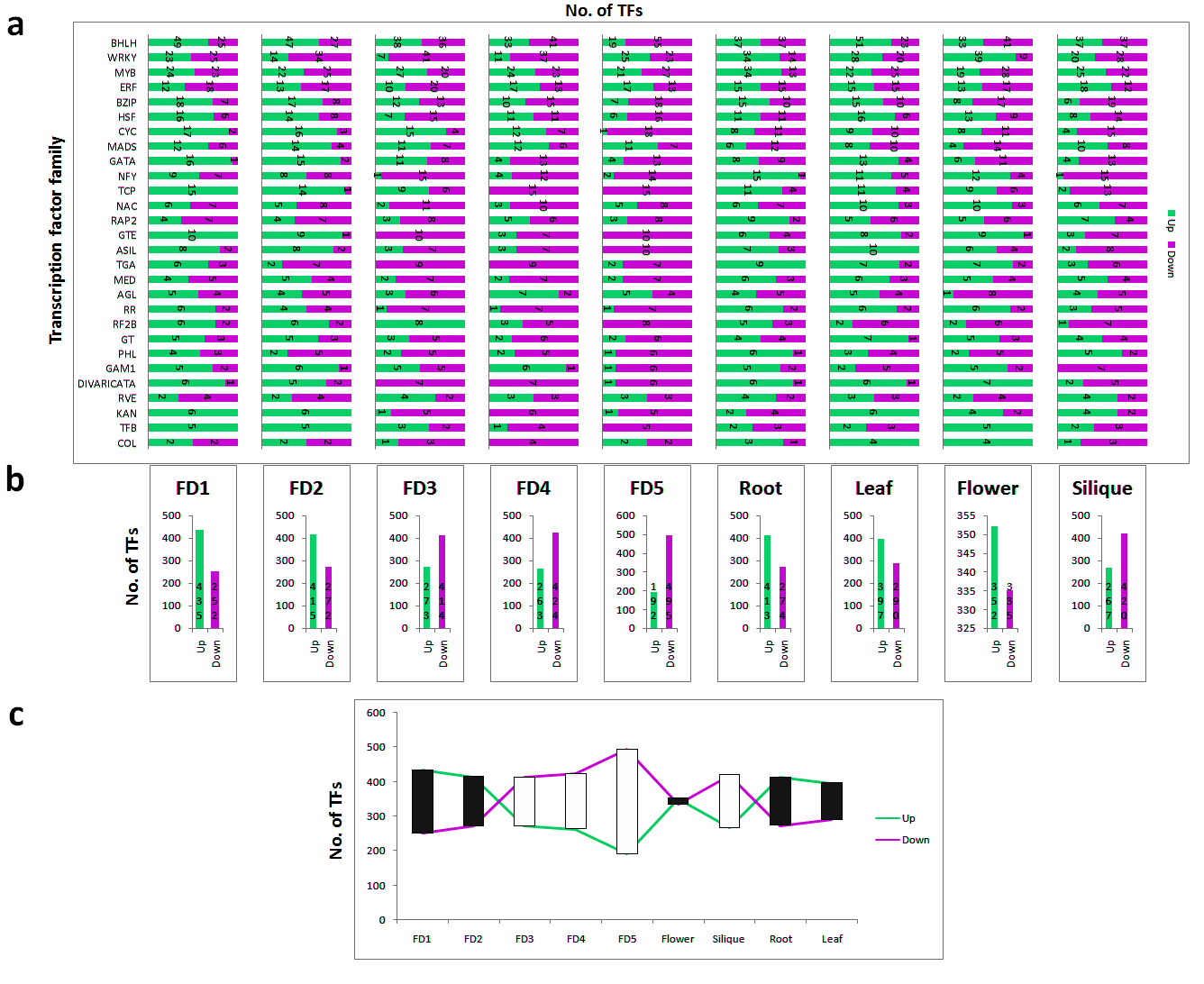
**

**Supplementary Figure 5** Search results of annotation of "Gibberellins" throughout the DEGs

**
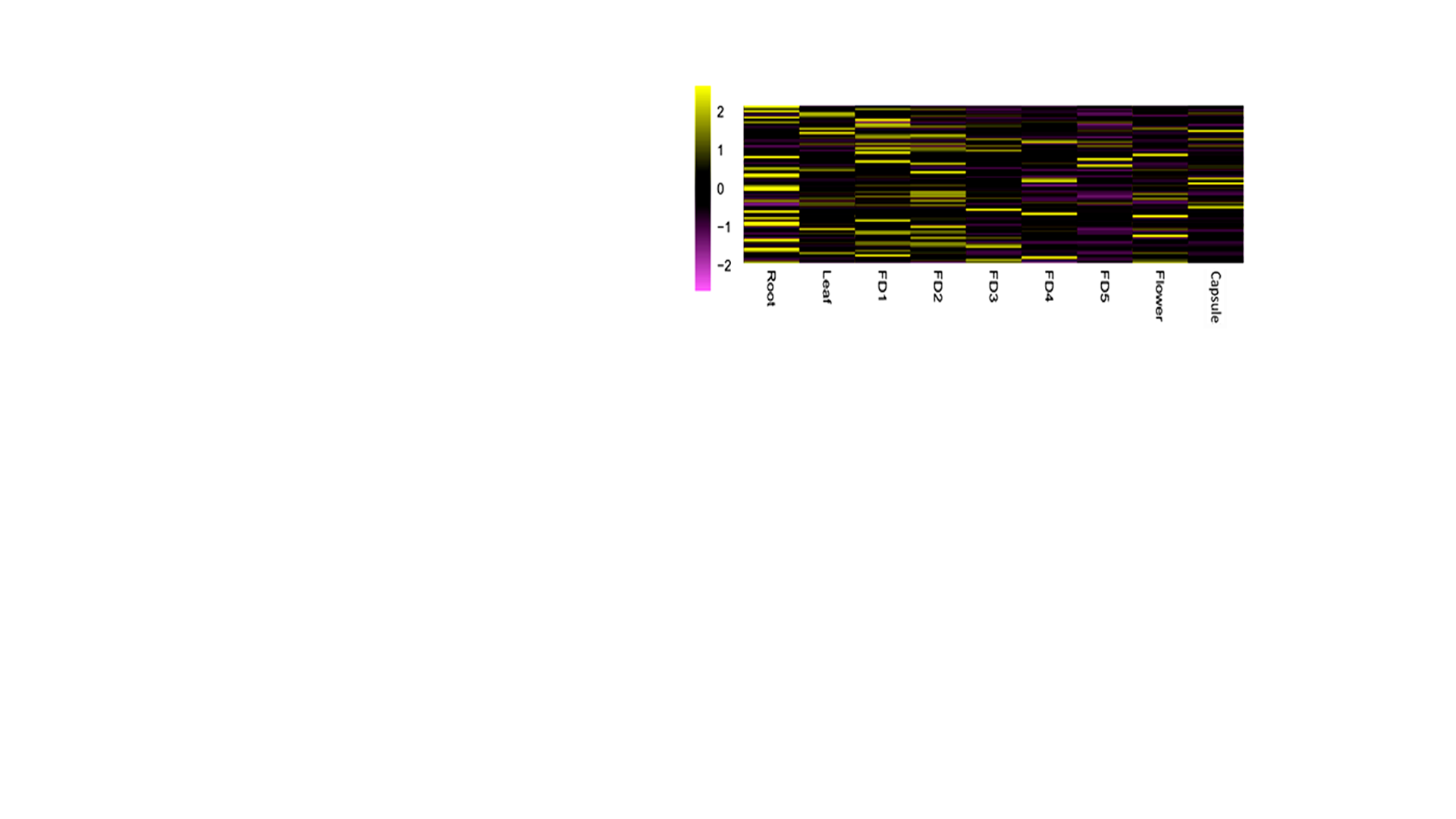
**
